# Supplementary material for: Novel Driver Strength Index highlights important cancer genes in TCGA PanCanAtlas patients
Source: PeerJ. 2022 Aug 11;10:e13860. doi: 10.7717/peerj.13860 (PMC9375969; doi:10.7717/peerj.13860)
Supplement: Supplemental Information 2 [file peerj-10-13860-s002.zip › COHORTS/genes plots/detailed/2021_8_16_14_9_distribution_events_detailed_females_13.pdf]

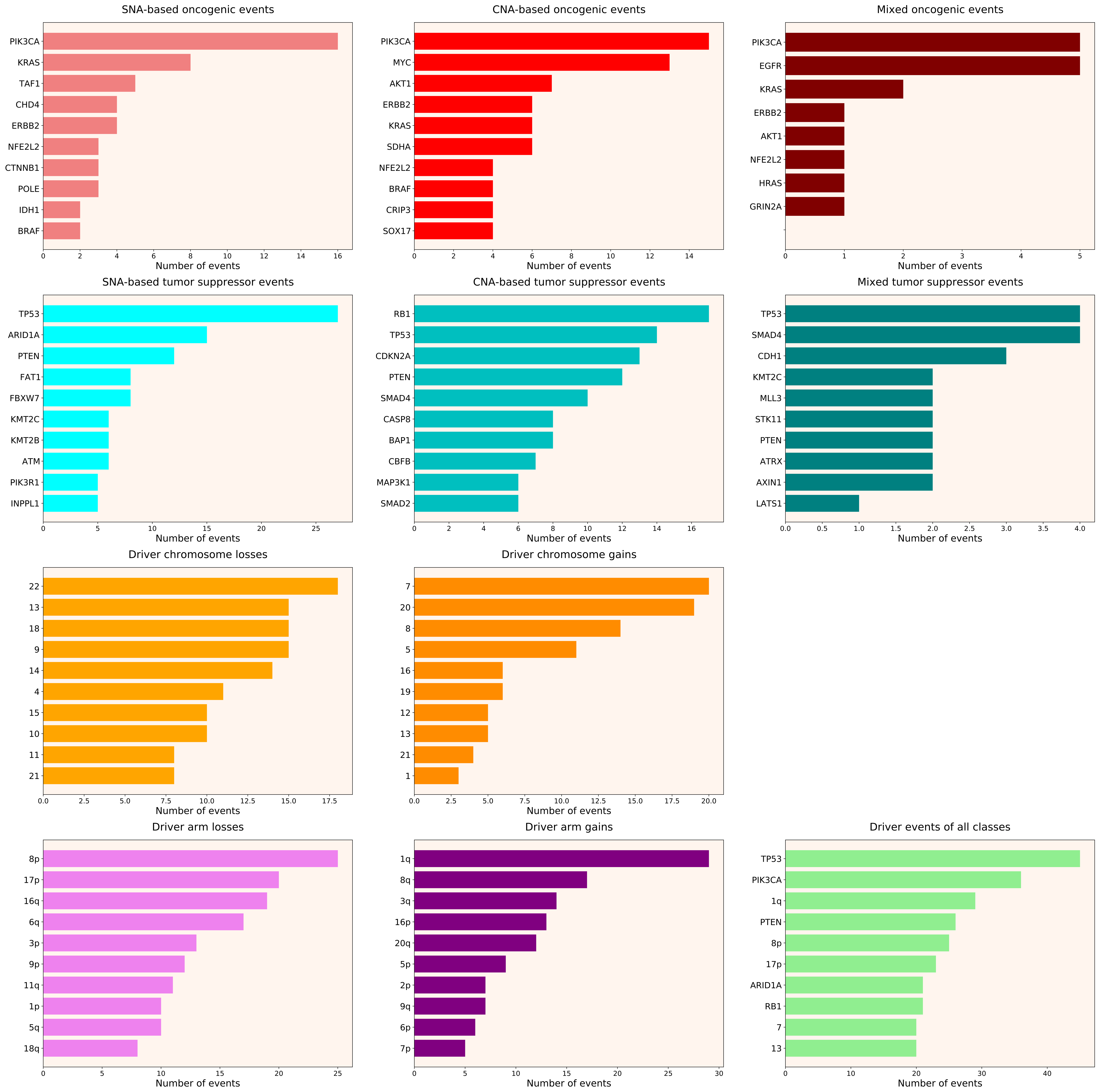

SNA-based oncogenic events
PIK3CA
KRAS
TAF1
CHD4
ERBB2
NFE2L2
CTNNB1
POLE
IDH1
BRAF
Number of events
CNA-based oncogenic events
PIK3CA
MYC
AKT1
ERBB2
KRAS
SDHA
NFE2L2
BRAF
CRIP3
SOX17
Number of events
Mixed oncogenic events
PIK3CA
EGFR
KRAS
ERBB2
AKT1
NFE2L2
HRAS
GRIN2A
Number of events
SNA-based tumor suppressor events
TP53
ARID1A
PTEN
FAT1
FBXW7
KMT2C
KMT2B
ATM
PIK3R1
INPPL1
Number of events
CNA-based tumor suppressor events
RB1
TP53
CDKN2A
PTEN
SMAD4
CASP8
BAP1
CBFB
MAP3K1
SMAD2
Number of events
Mixed tumor suppressor events
TP53
SMAD4
CDH1
KMT2C
MLL3
STK11
PTEN
ATRX
AXIN1
LATS1
Number of events
Driver chromosome losses
22
13
18
9
14
4
15
10
11
21
Number of events
Driver chromosome gains
7
20
8
5
16
19
12
13
21
1
Number of events
Driver arm losses
8p
17p
16q
6q
3p
9p
11q
1p
5q
18q
Number of events
Driver arm gains
1q
8q
3q
16p
20q
5p
2p
9q
6p
7p
Number of events
Driver events of all classes
TP53
PIK3CA
1q
PTEN
8p
17p
ARID1A
RB1
7
13
Number of events
